# Stress, Anxiety and Depression in students of a private medical school in Karachi, Pakistan

**DOI:** 10.12669/pjms.343.14664

**Published:** 2018

**Authors:** Noman Rehmani, Quratul Ain Khan, Syeda Sadia Fatima

**Affiliations:** 1 Noman Rehmani, MBBS Student. Medical College, Aga Khan University, Karachi, Pakistan; 2 Dr. Qurat ul Ain Khan, MD. Department of Psychiatry, Aga Khan University, Karachi, Pakistan; 3 Dr. Syeda Sadia Fatima, PhD. Department of Biological and Biomedical Sciences, Aga Khan University, Karachi, Pakistan

**Keywords:** Stress, Medical Education, Student mental health, University Students

## Abstract

**Objective:**

To determine frequency of stress, anxiety and depression and their coping mechanisms in undergraduate students of a private sector university.

**Methods:**

A cross sectional study was conducted at Aga Khan University recruiting students from Medical School, School of Nursing & Midwifery, and Dental Hygiene program who had attended at least six months on campus from October 2016 until August 2017. The “Aga Khan University Anxiety and Depression Scale” and “Student-Life Stress Inventory” scales were used to assess depression and anxiety, and stressors.

**Results:**

A total of 283 students participated in this study and all of them scored higher than the cutoff on both scales labeling them as highly stressed. Students from dental hygiene program reported more stressors as compared to MBBS (p<0.001) and SONAM (p=0.002). Factors identified as stressors included pressure to pass exam, meeting family’s expectations of good academic performance, and missing home.

**Conclusion:**

Stress, anxiety and depression are found to be highly prevalent among undergraduate students in medical setting in Karachi. Awareness, recognition, and timely management may reduce stress among the students and improve their performance and quality of life.

## INTRODUCTION

Stress is defined as the perception of discrepancy between environmental demands (stressors) and individual capacities to fulfill those demands. It occurs when an individual faces a situation that is perceived as over-whelming with which they cannot cope.^1^ Stress to a certain limit is known to enhance function and also sometimes referred to as favorable stress or eustress. When this limit exceeds and isn’t resolved by coping it is known as distress.^2^ Stress can cause or influence the course of both psychological conditions such as depression and anxiety and medical problems such as high blood pressure, poor wound healing etc.^3^ It is stated that anxiety is the psychophysiologic signal that the stress response has been initiated. Medical education in particular has become highly demanding and stressful posing a threat to the life of medical students. Increasing burden of workload compromises opportunities for students to relax or perform extracurricular activities. Studies have proved that medical students as compared to the general population are most distressed.^4^

The Aga Khan University (AKU) offers undergraduate programs in medicine, nursing and midwifery and allied health profession. These programs are tailored to meet the international standards in curriculum, student engagement and assessment. Great emphasis is given to the professional, personal and intellectual development of students. This is aimed to enable students to become aspiring medical professionals to deliver quality patient care, to advance healthcare policy and to make a difference in the communities of the developing world.

There are several sources of stress in medical school such as academic demands, time management, peer pressures, making important career choices, and financial constraints.^4^,^5^ Prevalence of stress in medical universities has been reported in many different countries and is estimated to be 41.9% in Malaysia, 31.2% in British universities, and 61.4% in Thailand.^6^-^8^ It is important that stress in undergraduate students is properly assessed and strategies are developed to help them cope with it. The aim of this study was to determine the frequency of stress, anxiety and depression in undergraduate students in a private sector university in Karachi and to identify stressors and coping mechanisms.

## METHODS

A cross sectional study was conducted at Aga Khan University (AKU) Karachi from October 2016 till August 2017. All students from year one to five of MBBS, year one to four of School of Nursing & Midwifery and year one to two of Diploma in dental hygiene who had attended at least six months of medical education on campus were recruited. Individuals who reported to be diagnosed with depression or taking treatment for any such condition were excluded from this study. The institutional ethical approval was obtained before data collection was commenced (Ref#4361-BBS-ERC-16). An email/SMS was circulated among the students informing about the study and its objectives. They were requested to sign a consent form if they agreed to participate in this research. They were informed that their participation was completely voluntary with a choice to remain anonymous. The questionnaires were distributed to the students by an independent researcher during the self-study time slots. Total time required to complete the two questionnaires was on an average 20 minutes. Biophysical and demographic profiles of the participants were collected such as age, weight, height, city of residence, year of education etc.

A pretested and validated scale called the “Aga Khan University Anxiety and Depression Scale” (AKUADS) was used to assess depression and anxiety. This scale has been developed specifically for the local population and consists of 25 items; 13 psychological and 12 somatic. The severity of symptoms is marked on a scale of 0 (never happens) to 3 (always happens) and 19 is considered a cut-off for anxiety with a specificity of 81%, sensitivity of 74%, a positive predictive value of 63%, and negative predictive value of 88%.^9^-^11^

Additionally, another validated self-reported questionnaire called “the Student-Life Stress Inventory” (SSI)^12^ was used to assess stressors. The SSI is a 51-item questionnaire, consisting of nine categories (five stressors and four reactions to stressors). The five stressors are: frustrations, conflicts, pressures, changes, and self-imposed. The four reactions to stressors were: physiological, emotional, behavioral, and cognitive appraisal. In responding to the SSI, participants must first indicate their overall view of stress as mild = 1, moderate = 2, or severe = 3. Then, they rate each of the 51 items on a 5-point Likert scale of 1 = never, 2 = seldom, 3 = occasionally, 4 = often, and 5 = most of the time. The values for each of the first eight categories were summed and recorded. The values for the last category (cognitive) were first reversed then summed and recorded. To obtain the total scores for the inventory, the recorded values for the nine categories were added. An open ended question was also included in the end to inquire about the stressors and coping strategies.

In order to achieve a power of 95% with a 15% estimated prevalence of depression/anxiety and a two-sided 5% level of significance, the minimum sample size required was n=196 (http://www.openepi.com/Menu/OE_Menu.htm); however we were able to recruit n=283 students for this study. Data were stored and analyzed using IBM-SPSS version 23.0, count and percentages were given for years of study, gender, and other qualitative parameters, mean and standard deviation were given for age, BMI, scores on scales (AKUADS and SSI) these scores were compared across Bachelor of Medicine and Bachelor of Surgery (MBBS), School of Nursing and Midwifery (SONAM) and Diploma in Dental Hygiene (DDH) students using one way ANOVA, and further post hoc analysis were done using Tukye’s test. Scores were compared between gender using independent sample t-test, spearman rank correlation was done to see the relationship of these scores with other factors, Pearson chi square test was done to see the association across MBBS, SONAM and DDH students, p-values less than 0.05 were considered significant.

## RESULTS

A total of 283 students participated in this study. BMI of all students was on average within normal weight category (Table-I). Majority of the students lived in hostels n=174 (61.48) while n=108 (38.3) were local resident and may explain higher rate of anxiety due to possible home sickness. Male to female ratio was similar in MBBS while more male students responded from SONAM and female students in DDH program.

**Table-I:** Demographics of participants.

Undergraduate Degree		Mean ± SD
MBBS (n=249)	Age (year)	20.91 ±1.99
	Weight (kg)	65.60 ± 11.91
	BMI (kg/m^2^)	22.50 ± 3.05
SONAM (n=15)	Age (year)	22.53 ± 1.80
	Weight (kg)	64.00 ± 5.91
	BMI (kg/m^2^)	22.09 ± 1.513
DDH (n=19)	Age (year)	20.26 ± 1.24
	Weight (kg)	57.32 ± 14.35
	BMI (kg/m^2^)	21.78 ± 4.44

The personal and family triggers for anxiety/ depression of the subjects is shown in Table-II.. Family history of depression was reported in 14% of medical school students; 26.3% of DDH students, while none from SONAM; where majority of them were seeking treatment for their ailments. In our study 41.1% MBBS; 53.3% SONAM and 21.1% DDH students reported to have failed in an exam.

**Table-II:** Personal and Family triggers for anxiety/ depression.

		Undergraduate Degree		

		MBBS	SONAM	DDH
Family history of depression	Yes	35 (14.0%)	0 (0.0)	5 (26.3)
Are the family members receiving treatment	Yes	20 (57.1)	0 (0.0)	3 (75.0)
Level of activity	Sedentary	44 (17.7)	4 (26.7)	4 (28.6)
	Walk 3 times/week	95 (38.2)	8 (53.3)	5 (35.7)
	Walk 5 times/week	110 (44.2)	3 (20.0)	5 (35.7)
Have you failed an exam	Yes	102 (41.1)	8 (53.3)	4 (21.1)

The AKUADS and SSI scores are sown in Table-III. All students scored >19 on AKUADS across all programs offered at AKU, which shows a higher stress/anxiety rate as compared to depression. Yet, when we asked the students if they thought that they had any symptoms of depression; 20% of medical school students, 40% of SONAM and 36.8% DDH students self-reported themselves as having mild depression. For the student stress response, we found the highest score for DDH students as compared to other degree programs (p=0.002). However, no significant difference was observed on AKUADS and SSI scores among years 1 to 5 of all Undergraduate Medical Education (UGME) programs. More females were found to be stressed and anxious as compared to males (x2= 9.678; p=0.008).

**Table-III:** AKUADS and SSI Scores of the study subjects.

AKUADS Score				

	Mean ± SD		p value	
MBBS	47.92 ± 5.97		0.145	
SONAM	50.80 ± 6.95			
DDH	46.89 ± 6.18			

**SSI Score**				

	**Stressor**	**Reaction to stressor**	**Total SSI**	**p value**

MBBS	57.48 ±15.14	57.95±16.38	115.43 ±29.21	0.188
SONAM	54.66 ±14.80	63.60±17.36	118.26 ±31.33^*^	0.000
DDH	63.42 ±15.23	76.10±21.02^*^	139.52±32.97^*^	0.003

**Gender wise Distribution**				

	**Male (n=144)**	**Female (n=139)**	**p value (T test)**	

AKUADS	43.70 ±5.45	49.93±9.16	0.003	
Stressor	57.17±14.81	58.08±15.41	0.612	
Reaction to Stressor	56.60±16.00	62.16±18.03	0.006	
Total SSI	113.77±28.57	120.25±30.90	0.068	

Significant difference was observed between girls and boys in facing situations and responding to stressors as girls showed higher levels of stress as compared to boys.

## DISCUSSION

Medical students go through higher levels of emotional and mental disturbance. They are expected to master a huge amount of knowledge and skill and they undergo regular pressures and the overwhelming burden barely leaves them with any time to relax. Along with the academic burden, they face a highly competitive environment that requires social and personal sacrifice.^4^,^13^-^15^ Our results show that all students scored >19 on AKUADS. This shows that all students have some element of stress and anxiety with varying severity. Several studies have shown high prevalence of anxiety among medical students in Pakistan ranging from 44%^16^, 45.5%^17^ to 60%^18^ and to as high as 74.2%.^19^ This study emphasizes the need for medical universities to develop strategies for recognition and management of stress in undergraduate medical students.

Majority of the students that participated in this study were either living in a hostel (58.9%) and/or with relatives. The higher rate of anxiety in these students can be explained by several possible factors. First years may have issues with adjusting in a place away from home, meeting new people and socializing. Furthermore, 14% of the students have a positive family history of depression that can also play an important contributor to the student’s wellbeing.

Twenty percent of the students reported that they felt depressed when asked in the study questionnaire; however this was identified when their forms were reviewed. Majority of these students were self-diagnosed and were not on any treatment. We later followed these subjects and referred them for counseling and appropriate treatment. Therefore the study helped identify if medical school was triggering their mental stability leading to outcomes that were worse than their baseline. Hence, these were students who perhaps had greater difficulty in combating the stressors compared to the rest.

The AKUADS and SSI scores were significantly higher in girls (49.9 and 120.25) than in boys (43.7 and 113.7). Another study from Pakistan showed that among female medical students 43.7% were anxious and 19.5% were depressed.^20^ Social attitudes and cultural norms tend to marginalize women in Pakistan and place a huge psychological impact on them. This can perhaps explain why there are more females suffering from stress, anxiety and depression compared to males. This trend could also be due to a different approach by females towards medical education in the sense of being competitive and securing higher marks in exams.

An interesting finding in our study was lack of significant difference in AKUADS and SSI scores between students of years one to five. Students in each year have some form of stress with different stressors present in each. Adjustment issues in first year, transitioning from theoretical to clinical learning in 3^rd^ year and graduation in final year are some stressors that can be identified. On the contrary, literature suggests that the prevalence of anxiety/depression increases proportionately throughout the academic years.^21^,^22^ A study conducted in Lahore emphasizes the fact that medical students feel stressed out due to lack of study-life balance, recreation, and sleep. These are attributed to the sense of competition and a large number of summative tests, fear of failure, scoring lower than hoped for, and high parental expectations.^13^ Our study differs from the previously published data in the sense that it focused on a versatile group of students in terms of degree program i.e. different modalities of medical education and was not limited to MBBS students only. Further, we not only assessed the prevalence but also were able to identify the stressors/ triggers and suggest valid coping mechanism/s. Most of the previous data used questionnaire related to depression in general, but we used an additional tool which is a specifically designed questionnaire for assessing stress in students.

The Aga Khan University has a state of the art Sports and Recreation Centre (SRC) that provides an excellent opportunity for students to sweat out all the stress that piles up after a busy schedule. SRC also provides a platform for other extracurricular activities comprising of training classes like yoga, musical instruments, fitness challenge etc. In addition, the university encourages the student body to form committees for multiple activities each of which has a representative from every batch. Some of these include Arts and Culture Committee (ACC), Sports, Literature, Curriculum and Students lounge etc. where the students organize several events throughout the year. More importantly all academic committees also have a student representative that helps provide a platform for students to voice their opinions and any disagreements that they may have with the curriculum or exam standards.

When we enquired our students about the coping strategy and the utility of student counselor for solving such issues; majority responded that initially they felt shy to avail help. But as the academic years progressed, it became clearer to them to seek help and contact the student mentors, faculty mentors or psychologists assigned by the University for catering to their needs. Yet, they still believe that stress management and study skill management sessions should be regularly organized throughout the years and believe that these may help reduce anxiety. AKU has provided multiple highly qualified student counselors for all those who have been unsuccessful in tackling their daily life struggle and still feel under pressure due to the highly competitive environment. Students commented that being a part of the best medical university of Pakistan; they feel that there is a cut throat competition to get honors and scholarships and simultaneously manage time for preparing for their board exams such as USMLE’s. These stresses coupled with a busy working schedule, particularly in the clinical years, leaves little time to relax.

## RECOMMENDATIONS

Strategies are needed to target high levels of stress in undergraduate medical students. Sports, yoga and other physical activities should be encouraged to tackle stress along with proper eating and sleeping habits. To deal with fear and anxiety of examinations, regular assignments should be given and mock examinations should be taken that can help them prepare for the actual exam. For a long-term benefit, counseling should be started at an early stage and should be an integral part of the academic curriculum. With the help of these strategies, psychological problems will be detected earlier and the management will reduce undue stress and will have less social impairment in future.^8^

## CONCLUSION

Stress, anxiety and depression are found to be highly prevalent among undergraduate students in medical setting in Karachi especially in association with certain stressors. Awareness, recognition, and timely management may reduce stress among the students and improve their performance and quality of life.
